# Association between number of institutions with coronary computed tomography angiography and regional mortality ratio of acute myocardial infarction: a nationwide ecological study using a spatial Bayesian model

**DOI:** 10.1186/s12942-018-0133-0

**Published:** 2018-05-21

**Authors:** Hideaki Kawaguchi, Soichi Koike, Ryota Sakurai, Kazuhiko Ohe

**Affiliations:** 1 Department of Biomedical Informatics, The University of Tokyo, 7-3-1 Hongo, Bunkyo, Tokyo, 113-0033 Japan; 2 Division of Health Policy and Management, Center for Community Medicine, Jichi Medical University, 3311-1 Yakushiji, Shimotsuke, Tochigi, 329-0498 Japan

**Keywords:** Coronary computed tomography angiography, Acute myocardial infarction, Standardized mortality ratio, Health services research, Healthcare access

## Abstract

**Background:**

Coronary computed tomography angiography (CTA) has demonstrated high diagnostic accuracy for detection of coronary artery stenosis, and healthcare providers can detect coronary artery disease in earlier stages before it develops into more serious clinical conditions such as acute myocardial infarction (AMI). We hypothesized that the mortality ratio of AMI in regions with a higher density of coronary CTA is lower than that in regions with a lower density of coronary CTA.

**Methods:**

This ecological and cross-sectional study using secondary data targeted all secondary medical service areas (SMSAs) in Japan (n = 349). We obtained the numbers of cardiologists, institutions with coronary CTA, and institutions with a cardiac catheterization laboratory (CCL) as medical resources, socioeconomic factors, lifestyle factors, exercise habit factors, and AMI mortality data from a Japanese national database. We evaluated the association between the number of these medical resources and the standardized mortality ratio (SMR) of AMI in each SMSA using a hierarchical Bayesian model accounting for spatial autocorrelation (i.e., a conditional autoregressive model). We assumed a Poisson distribution for the observed number of AMI-related deaths and set the expected number of AMI-related deaths as the offset variable.

**Results:**

The number of institutions with coronary CTA was negatively and significantly associated with the SMR of AMI (relative risk [RR] 0.900; 95% credible interval [CI] 0.848–0.953), while the SMR in each SMSA was not significantly associated with the number of either cardiologists (RR 0.997; 95% CI 0.988–1.004) or institutions with a CCL (RR 1.026; 95% CI 0.963–1.096).

**Conclusions:**

We observed a significant association between the number of institutions with coronary CTA and the SMR of AMI. Effective allocation of coronary CTA in each region is recommended, and it would be important to clarify the standing position of coronary CTA in regional networking for AMI treatment in the future.

**Electronic supplementary material:**

The online version of this article (10.1186/s12942-018-0133-0) contains supplementary material, which is available to authorized users.

## Background

Coronary artery disease (CAD) is the leading cause of death worldwide [1]. Coronary artery disease caused 7.2 million deaths in 2008 and 7.4 million deaths in 2012, which exceeded 10% of the total global mortality [1, 2]. In Japan, CAD was the second-highest cause of death in 2015 [3], and the number of CAD-induced deaths in Japan was the third highest among high-income countries [1]. Therefore, both primary and secondary prevention of CAD is of great importance.

During the past decade, the diagnostic accuracy of coronary computed tomography angiography (CTA), which is a noninvasive and anatomical imaging technique for detection of CAD, has been improved [4–6]. Using coronary CTA, health providers can detect preclinical CAD in advance and administer treatment before the development of a more critical condition such as acute myocardial infarction (AMI). For example, in the updated National Institute for Health and Care Excellence (NICE) guidelines, coronary CTA is the first-line investigation for patients presenting with new-onset chest pain due to suspected CAD [7]. The updated NICE guideline CG95 poses a far wider and more uniform geographical delivery of coronary CTA as a major challenge [8]. Considering that construction of regional networks is internationally recommended for treatment of AMI [9], evidence of how to allocate coronary CTA in each regional is important. Moreover, from the viewpoint of public health, appropriate allocation of coronary CTA seems of great importance to efficiently reduce the number of AMI-related deaths in each region. However, no study has evaluated the association between the geographical distribution of coronary CTA and AMI-related death by region. We hypothesized that the mortality ratio of AMI in regions with a higher density of coronary CTA would be lower than that in regions with a lower density of coronary CTA. In the present study, we evaluated the nationwide association between the geographical distribution of coronary CTA and the mortality ratio of AMI.

## Methods

### Study design

This was an observational, cross-sectional study using secondary data. This study was also a nationwide ecological study targeting the whole of Japan. Japan comprises 47 prefectures, and the Japanese government established subprefectural medical regions called secondary medical service areas (SMSAs) [10]. An SMSA is defined as a medical unit that evaluates demand and supply of health resources for inpatient treatment, especially hospital care. We targeted all SMSAs in Japan according to a survey in 2011 (n = 349).

### Data source

Geographical information, such as municipality boundary data, was obtained from the Municipality Map Maker for Web [11]. We combined each municipality-level parameter to form the SMSA data because each SMSA consists of several municipalities. We used ArcGIS version 10.2.1 (ESRI Japan Inc., Tokyo, Japan).

The Japan Ministry of Health, Labour and Welfare (MHLW) conducts a biennial census survey of the working conditions of all physicians, dentists, and pharmacists. Details from this census survey were used to obtain the number of self-reported cardiologists in each SMSA. The Japan MHLW also conducts a detailed triennial survey of all medical institutions. The data from this survey were used to obtain the number of institutions with coronary CTA and the number with a cardiac catheterization laboratory (CCL) in each SMSA. Because of the survey questionnaire style, the institutions with coronary CTA included those with cardiac magnetic resonance imaging (MRI). However, we assumed that almost all institutions with cardiac MRI had also coronary CTA when compared with another data source from the Japanese Circulation Society [12]. In addition, we regarded institutions with cardiac digital radiography as those with a CCL. We obtained permission from the MHLW to analyze these survey data. Both survey forms are available on the MHLW website [13].

Data regarding socioeconomics, lifestyle, and exercise habits were obtained from the national databases of the following surveys: Population Census, Survey of municipal tax situation, School Basic Survey, Comprehensive Survey of Living Conditions, and National Health and Nutrition Survey. All data are available from the national Japanese portal site, e-Stat [14].

The AMI mortality data were obtained from the official database of Vital Statistics, which covers all deaths in Japan in principle. In this study, we obtained the number of deaths classified by the International Classification of Diseases-10 codes I21 (AMI) and I22 (subsequent myocardial infarction).

### Explanatory variables

We used the numbers of cardiologists, institutions with coronary CTA, and institutions with a CCL per 100,000 population. As socioeconomic covariates, we used available socioeconomic factors at an SMSA level from e-Stat that had been identified in previous studies [10, 15, 16]. These factors were the population density (people per hectare), average per capita income (in 1000 JYN), unemployment rate (%), proportion of university graduates per population aged > 15 years (%), and divorce rate (incidence per 1000 population). Because the population density distribution was extremely skewed (skewness, 3.12; kurtosis, 13.4), we took a logarithm of that variable. As covariates of lifestyle and exercise habits, we used data on smoking habits, drinking habits, and the estimated body mass index as lifestyle data and the average number of daily steps as the exercise habit datum. We defined a drinker as a person who drank at a frequency of “not less than 1 to 3 days per month,” and we defined a smoker as a person who “occasionally smoked.” Because a prefecture was the smallest unit serving as the target area for lifestyle and exercise habit data, these data at an SMSA level in the same prefecture took the same value.

Since the year in which these surveys are conducted differs, we obtained the data for the numbers of institutions with coronary CTA and with a CCL from the fiscal year 2011, the data for drinking habits from the fiscal year 2013, and all other data from the fiscal year 2010.

### Statistical analysis

#### Outcomes

We calculated the ratios for AMI mortality based on standard mortality ratios (SMRs). The SMR is calculated by dividing the observed number by the expected number of AMI-related deaths. We calculated the ratios separately for men and women and for eight age subgroups: < 20, 21–30, 31–40, 41–50, 51–60, 61–70, 71–80, and > 80 years. We calculated the national AMI mortality rates in each subgroup, then multiplied the national AMI mortality rates by the population of each subgroup in each SMSA to obtain the expected number of AMI-related deaths.

#### Statistical model

Typically, when we handle spatial data, there is likely to be a correlation relating to the location of each region; i.e., a spatial autocorrelation. Moran’s I statistic was selected to quantify the degree of spatial autocorrelation. To model the spatial autocorrelation, we used a conditional autoregressive (CAR) model, which is a hierarchical Bayesian model [17]. In the CAR model, random effects are set as error terms in general regression models by the class of CAR prior distribution, which is a type of Markov random field model. More concretely, random effects are represented by a set of full conditional distributions using a binary neighborhood matrix. If two regions are considered to be neighbors, their random effects are correlated. Additional details on the CAR model have been previously published [18]. Among several kinds of CAR models, we chose the CAR Leroux model, which was reported in a simulation-based study to be consistently suitable for various spatial correlation scenarios [18]. A Poisson distribution was assumed for the observed number of AMI-related deaths, and we set the expected number of AMI-related deaths as the offset variable in the CAR Leroux model. We used Markov chain Monte Carlo (MCMC) simulations with 110,000 iterations; the first 10,000 iterations were discarded as burn-in. We used Geweke’s diagnostic to check MCMC convergence [19]. We estimated the relative risk (RR) and 95% Bayesian credible interval (CI) of each variable. According to a previous study [20], we considered that an association is not significant if the 95% CI of RR includes 1.

We used the widely applicable information criterion (WAIC) as a measure of the goodness-of-fit of the Bayesian statistical model; a model with a lower WAIC was considered the better-fit model [21]. We compared the CAR Leroux model with a normal Poisson regression model without consideration for spatial autocorrelation using the WAIC.

We evaluated multicollinearity of covariates using the variance inflation factor (VIF) [22]. Although the proportions of university graduates and the average per capita income were important factors, we removed them because they had the greatest VIF. All other variables had a VIF of < 2.5 and were entered into the CAR Leroux model.

Descriptive statistics are shown as median and interquartile range, and all analyses were conducted using R V.3.2.4 (https://www.r-project.org) [23].

## Results

### Descriptive statistics

**Fig. 1:**
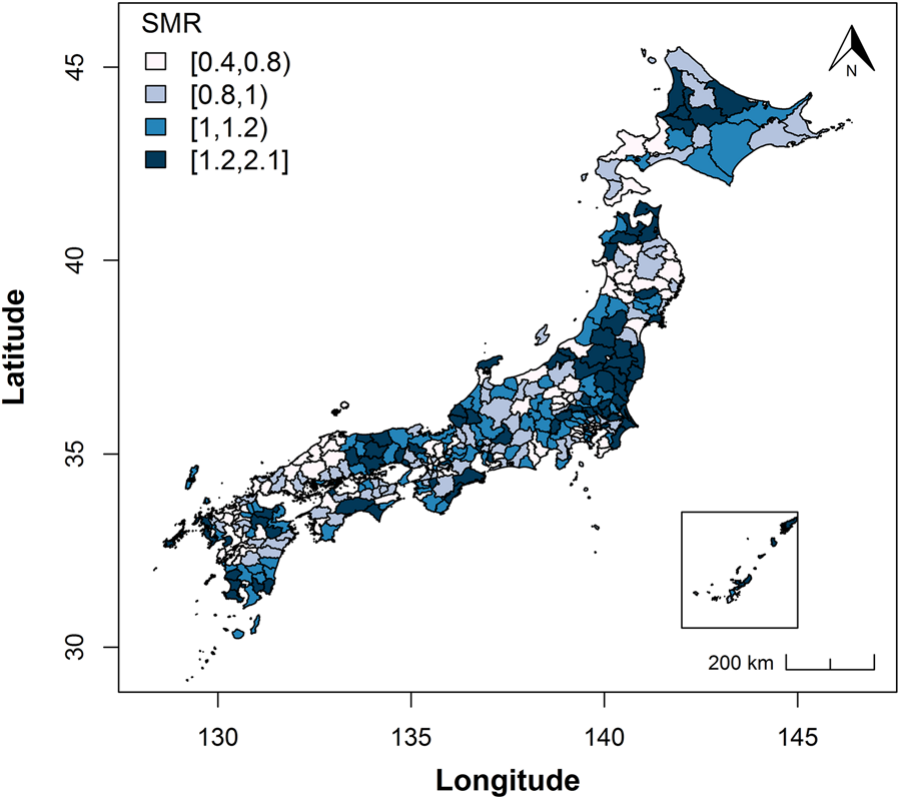
SMR of acute myocardial infarction. The dark blue regions have the highest SMR. SMR, standard mortality ratio

**Fig. 2:**
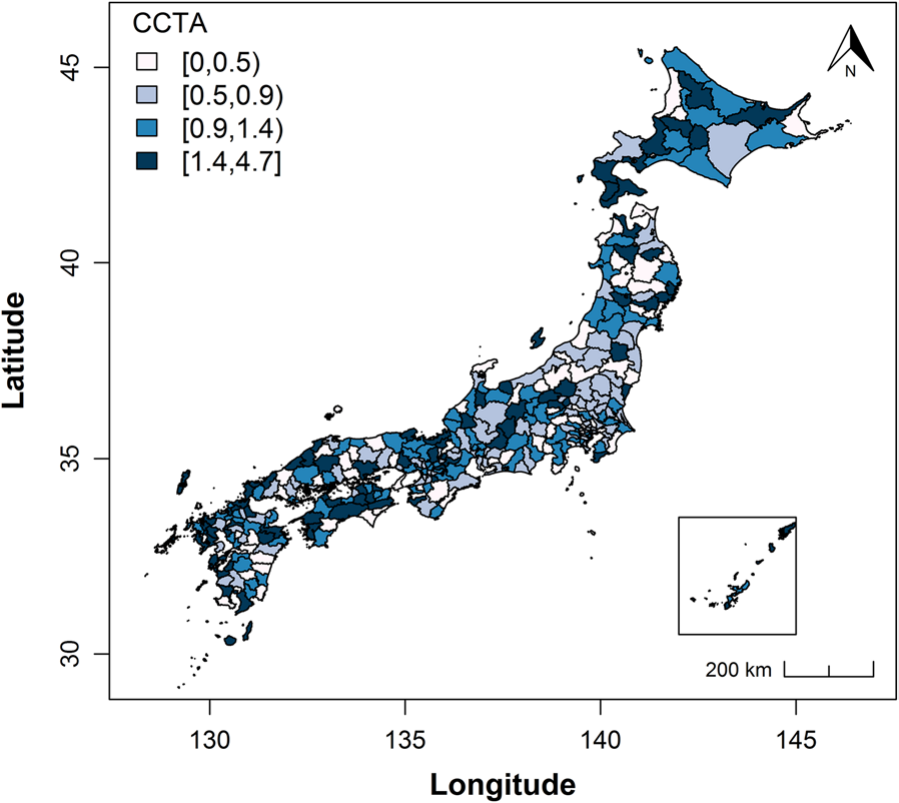
Number of institutions with coronary CTA per 100,000 population. The dark blue regions have the highest number of institutions. CTA, computed tomography angiography

The mean and standard deviation of the SMR of AMI were 1.039 and 0.313, respectively, indicating a wide range across SMSAs. Figure 1 shows the distribution of the SMR of AMI across SMSAs in Japan in 2011. Moran’s I statistic for the SMR was 0.306 (p < 0.001), which indicates strong spatial autocorrelation of the SMR in Japan. Figure 2 shows the number of institutions with coronary CTA per 100,000 population. Moran’s I statistic for the institutions with coronary CTA was 0.053 (p = 0.03), which indicates moderate and statistically significant spatial autocorrelation.

Table 1 shows descriptive data for the 349 SMSAs included in this study. The third quantiles of the numbers of all three medical resources (i.e., cardiologists, institutions with coronary CTA, and institutions with a CCL) were more than twice the first quantiles of those variables. This result indicates that these medical resources varied across SMSAs.

**Table 1:** Characteristics of 349 secondary medical service areas in Japan

	Data source	Median [IQR]
SMR of AMI	Vital Statistics	0.985 [0.807–1.187]
Cardiologists per 100,000 population (persons)	Survey of Physicians, Dentists and Pharmacists	6.274 [4.180–9.142]
Institutions with coronary CTA per 100,000 population (institutions)	Survey of Medical Institutions	0.926 [0.546–1.379]
Institutions with a CCL per 100,000 population (institutions)	Survey of Medical Institutions	0.925 [0.571–1.332]
Logarithm of population density per hectare	Population Census	1.969 [1.378–2.658]
Unemployment rate (%)	Population Census	6.350 [5.529–7.167]
Divorce rate (incidence per 1000 population)	Population Census	1.851 [1.641–2.028]
Average per capita income in 1000 JYN	Survey of municipal tax situation	2750 [2546–3050]
Proportion of university graduates (%)	School Basic Survey	12.04 [8.78–16.20]
Body mass index	National Health and Nutrition Survey	23.12 [22.85–23.44]
One-tenth of average number of daily steps (steps)	National Health and Nutrition Survey	716.3 [680.0–749.5]
Smoking habit (%)	Comprehensive Survey of Living Conditions	19.11 [17.98–20.53]
Drinking habit (%)	Comprehensive Survey of Living Conditions	42.90 [40.42–43.91]

*IQR* interquartile range, *SMR* standard mortality ratio, *AMI* acute myocardial infarction, *CTA* computed tomography angiography, *CCL* cardiac catheterization laboratory

### Results of CAR Leroux model

Table 2 shows the detailed results of the CAR Leroux model. The absolute value of Geweke’s diagnostic for each parameter was < 1.96, which means that all parameters used in the MCMC simulations converged [19].

**Table 2:** Results of estimating conditional autoregressive Leroux model for acute myocardial infarction mortality ratio

	Median	2.50%	97.50%
(Intercept)	0.540	0.019	10.73
Cardiologists per 100,000 population	0.997	0.988	1.004
Institutions with coronary CTA	0.900	0.848	0.953
Institutions with a CCL	1.026	0.963	1.096
Log(population density)	0.950	0.901	1.003
Unemployment rate	1.017	0.986	1.048
Divorce rate	0.949	0.824	1.089
Body mass index	1.045	0.930	1.190
One-tenth of average number of daily steps	1.001	0.9998	1.002
Smoking habit	1.035	1.002	1.069
Drinking habit	0.963	0.945	0.980
ρ	0.444	0.217	0.761

Numbers in this table except for ρ are shown as relative risks. ρ, ranging from 0 to 1, indicates the strength of spatial autocorrelation

*CTA* computed tomography angiography, *CCL* cardiac catheterization laboratory

Table 2 shows the significantly negative association between the number of institutions with coronary CTA and the SMR of AMI (RR = 0.900, 95% CI = 0.848–0.953). Across Japan, an increase of 1300 institutions with coronary CTA was associated with a 10% decrease in the SMR of AMI. In contrast, the SMR in SMSAs was not significantly associated with the number of either cardiologists or institutions with a CCL. A smoking habit was positively associated with the SMR of AMI, while a drinking habit was negatively associated. All other socioeconomic factors were not significantly associated with the SMR.

The WAIC was 5683.486 for the normal Poisson regression model without consideration for spatial autocorrelation and 2791.118 for the CAR Leroux model. Because the model with the lower WAIC was considered the better fit, this result indicates that the CAR Leroux model was much better fit than the normal Poisson regression model.

## Discussion

The main finding of this nationwide ecological study is the significant geographical association between the number of institutions with coronary CTA and the SMR of AMI. To the best of our knowledge, the current study is the first to clarify this association. This association can be said to be an important international finding in that it directly focuses on both coronary CTA allocation and regional clinical outcomes, considering that allocation of coronary CTA is receiving more attention in countries beyond Japan as in the updated NICE guidelines [7, 8]. This finding was obtained because of spatial statistics; application of the method to the topic is also a novelty of the current study.

Two major randomized controlled trials, PROMISE [24] and SCOT-HEART [25], showed several effects of coronary CTA on clinical outcomes. The former reported a reduction in death or nonfatal myocardial infarction in the coronary CTA group over the initial 12-month follow-up period (hazard ratio, 0.66; p = 0.049) compared with the functional testing group, although this difference was not recognized at the end of follow-up. The latter reported that the use of coronary CTA led to a 38% reduction in coronary heart disease-related death and nonfatal myocardial infarction over a median follow-up of 1.7 years compared with standard care alone. Although further investigations will be required to elucidate the association between coronary CTA and clinical outcomes [26], the current study demonstrated an impact of coronary CTA on clinical outcomes, particularly for AMI mortality, from the viewpoint of an ecological association.

The results of the current study suggest an important role of coronary CTA in specialized regionalized network systems. Compared with CCL, coronary CTA is easy to establish in rural medical areas and has been expected to be a gatekeeper for stratifying CAD patients [27]. Considering this role of coronary CTA, how to allocate coronary CTA in each region is becoming increasingly more important. The construction of specific regional networks is reportedly essential for timely AMI treatment, particularly percutaneous coronary intervention [28–31]. There is a trend of treating AMI on regional networks worldwide [9]. In contrast, the standing position of coronary CTA, a relatively new technique, remains unclear in regional networks. The findings of this study suggest that effective allocation of coronary CTA in regionalized networks would be important to improve regional outcomes for AMI. For example, it has been proposed that smaller facilities could be used as triage points [32], and coronary CTA equipment in these facilities might enhance triage performance.

Prediction of the future geographical distribution of coronary CTA is also important to effectively allocate coronary CTA. The spatial competition model would be helpful for this prediction [33]. According to this model, even if the distribution of service resources is concentrated in cities with large populations for maximal profit, relocation of these resources toward smaller cities can occur because of the increasingly keener competition for profit in large cities. This model could be applied to medical devices such as CT, MRI, and positron emission tomography [34]. Because coronary CTA is relatively new medical technique, its maldistribution could be improved in the future. For example, the spread of coronary CTA is still insufficient in the United Kingdom, and the maldistribution might be improved with an increase in coronary CTA [8]. The frequency of using CT is higher in Japan than in other countries [35], and the results in Japan might be helpful for future allocation of coronary CTA in countries where coronary CTA is not sufficient.

While the number of institutions with coronary CTA was associated with the SMR, the number of institutions with a CCL was not significantly associated with the SMR. Considering that the number of coronary angiography (CAG) varies among medical institutions, we also evaluated the association between the number of CAG in each SMSA and the SMR of AMI. We found no significant association between them [see Additional file 1: Table A1]. This result could stem from the recent trend in which percutaneous coronary intervention is performed not only for surviving patients but also to improve patients’ quality of life, particularly patients with stable CAD as shown in the COURAGE trial [36].

The number of cardiologists was not significantly associated with the SMR of AMI. This result is consistent with a previous study in Japan [10]. In contrast, however, a previous study in the US showed that the mortality risk in patients hospitalized for AMI in regions with a low density of cardiologists was higher than in regions with a high density of cardiologists [15]. The discordance between our results and the study in the US may have two explanations. First, while the study in the US used patient-level data, the current study was based on SMSA-level data. Because of the difference in sample sizes, the previous study had higher statistical power to detect associations than did our study. Second, the previous study did not consider the influence of medical devices, particularly coronary CTA. As an additional analysis, we constructed a Bayesian model that excluded the number of institutions with coronary CTA and institutions with a CCL [see Additional file 1: Table A2]. This model showed a greater tendency toward a statistically significant negative association between the number of cardiologists and the SMR of AMI (RR 0.993; 95% CI 0.986–1.001) than the results shown in Table 2 (RR 0.997; 95% CI 0.988–1.004). If the previous study had considered the influence of coronary CTA, the association might be not significant.

One of the biggest advantages of the present study is its consideration of the spatial autocorrelation. Ignoring this parameter introduces a risk of incorrect statistical model estimation, particularly a risk of false-positive significance [37]. As shown in Table 2, the posterior distribution of the spatial autocorrelation exhibited a moderate spatial autocorrelation (ρ 0.444; 95% CI 0.217–0.761). Given that the WAIC of the CAR Leroux model was approximately half that of the normal Poisson regression model, we should explicitly use spatial autocorrelation in statistical models that handle spatial data. This advantage of our approach is expandable in that it is applicable not only to the distribution of coronary CTA but also to the distribution of any medical resources.

## Limitations

This study has several limitations. First, it was an ecological analysis and did not account for patient-level factors that might contribute to AMI-related death, such as comorbidities, the severity of AMI in each patient, and other personal factors, resulting in possible ecological fallacy. Future researchers should also collect and analyze microdata regarding AMI-related deaths. Second, this study had a risk of information bias derived from the use of secondary data sources. For example, based on the survey questionnaire style, the number of institutions with coronary CTA included the number of institutions with cardiac MRI. In addition, we obtained the data regarding cardiologists by counting the number of cardiologists whose main medical department was “cardiology.” However, because of the questionnaire format, there might have been several physicians who responded that they were “internists” despite the fact that they were engaged in cardiology. Third, the results of this study might not be applicable to other countries. Notably, the frequency of using CT is higher in Japan than in other countries [35]. In addition, because using CT is popular in Japan, much fewer institutions tend to perform functional stress testing compared to coronary CTA [12], while functional stress testing is a major non-invasive testing especially in the US. Therefore, we could not obtain detailed data of the number of functional stress testing institutions. Further data from other countries should be used to verify the generalizability of the results in this study.

Despite these limitations, this study provides the first evidence of an association between the geographical distribution of coronary CTA and regional AMI-related deaths. To the best of our knowledge, no other studies have assessed this association, and our findings will therefore help the government to determine how to efficiently allocate coronary CTA. We have enhanced the study’s accuracy by using spatial statistical models to consider the spatial autocorrelation.

## Conclusions

The number of institutions with coronary CTA was significantly negatively associated with the SMR of AMI, while the SMR in each SMSA was not significantly associated with the number of either cardiologists or institutions with a CCL. Proactive allocation of coronary CTA is recommended, and it would be important to clarify the standing position of coronary CTA in regional networking for AMI treatment in the future.

## Additional file

**Additional file 1.** Results of additional experiments supporting our discussions.

## Abbreviations

AMI: acute myocardial infarction
CAD: coronary artery disease
CAG: coronary angiography
CAR: conditional autoregressive
CCL: cardiac catheterization laboratory
CI: credible interval
CTA: computed tomography angiography
MCMC: Markov chain Monte Carlo
MHLW: Ministry of Health, Labour and Welfare
MRI: magnetic resonance imaging
RR: relative risk
SMR: standardized mortality ratio
SMSA: secondary medical service area
VIF: variance inflation factor
WAIC: widely applicable information criterion
