# Supplementary material for: Association between number of institutions with coronary computed tomography angiography and regional mortality ratio of acute myocardial infarction: a nationwide ecological study using a spatial Bayesian model
Source: Int J Health Geogr. 2018 May 21;17:13. doi: 10.1186/s12942-018-0133-0 (PMC5963166; doi:10.1186/s12942-018-0133-0)
Supplement: Supplementary file 1 — Additional file 1. Results of additional experiments supporting our discussions. [file 12942_2018_133_MOESM1_ESM.pdf]

Additional file 1

Table A1. Results of estimating conditional autoregressive Leroux model for acute myocardial infarction mortality ratio in which the number of institutions with a cardiac catheterization laboratory was exchanged for the number of catheterization tests. All numbers in this table except for ρ are shown as relative risk. ρ, ranging from 0 to 1, indicates the strength of the spatial autocorrelation.

	Median	2.50%	97.50%
(Intercept)	0.543	0.023	10.761
Cardiologists per 100,000 population	0.995	0.987	1.004
Institutions with coronary CTA	0.909	0.867	0.955
Number of CAG	1.0004	0.9995	1.001
Log(population density)	0.949	0.892	1.001
Unemployment rate	1.017	0.988	1.048
Divorce rate	0.958	0.836	1.106
Body mass index	1.045	0.931	1.187
One-tenth of average number of daily steps	1.001	0.9996	1.002
Smoking habit	1.034	1.004	1.068
Drinking habit	0.963	0.946	0.982
ρ	0.456	0.219	0.776

CTA, computed tomography angiography; CAG, coronary angiography

Table A2. Results of estimating conditional autoregressive Leroux model for acute myocardial infarction mortality ratio, which excluded the number of institutions with coronary computed tomography angiography and a cardiac catheterization laboratory from the model constructed in the main text. All numbers in this table except for ρ are shown as relative risk. ρ, ranging from 0 to 1, indicates the strength of the spatial autocorrelation.

	Median	2.50%	97.50%
(Intercept)	0.278	0.011	5.974
Cardiologists per 100,000 population	0.993	0.986	1.001
Log(population density)	0.953	0.904	1.003
Unemployment rate	1.011	0.979	1.044
Divorce rate	0.980	0.839	1.131
Body mass index	1.062	0.946	1.201
One-tenth of average number of daily steps	1.001	0.9999	1.002
Smoking habit	1.034	1.004	1.067
Drinking habit	0.965	0.949	0.982
ρ	0.417	0.194	0.739
